# Supplementary material for: Characterization of tumor evolution by functional clonality and phylogenetics in hepatocellular carcinoma
Source: Commun Biol. 2024 Mar 29;7:383. doi: 10.1038/s42003-024-06040-9 (PMC11245610; doi:10.1038/s42003-024-06040-9)
Supplement: Supplementary file 2 — Supplementary Information [file 42003_2024_6040_MOESM2_ESM.pdf]

Supplementary Information

Characterization of tumor evolution by functional clonality and phylogenetics in hepatocellular carcinoma

Zeynep Kacar, Eric Slud, Doron Levy, Julián Candia, Anuradha Budhu, Marshonna Forgues, Xiaolin Wu, Arati Raziuddin[5], Bao Tran[5], Jyoti Shetty[5], Yotsawat Pomyen[6], Jittiporn Chaisaingmongkol[6], Siritida Rabibhadana[6], Benjarath Pupacdi[6], Vajarabhongsa Bhudhisawasdi[7], Nirush Lertprasertsuke[8], Chirayu Auewarakul[9], Suleeporn Sangrajrang[10], Chulabhorn Mahidol[6], Mathuros Ruchirawat[6,11], Xin Wei Wang[1, 4, *]

This file contains:

Supplementary Figures 1-10
Supplementary Tables 1-12

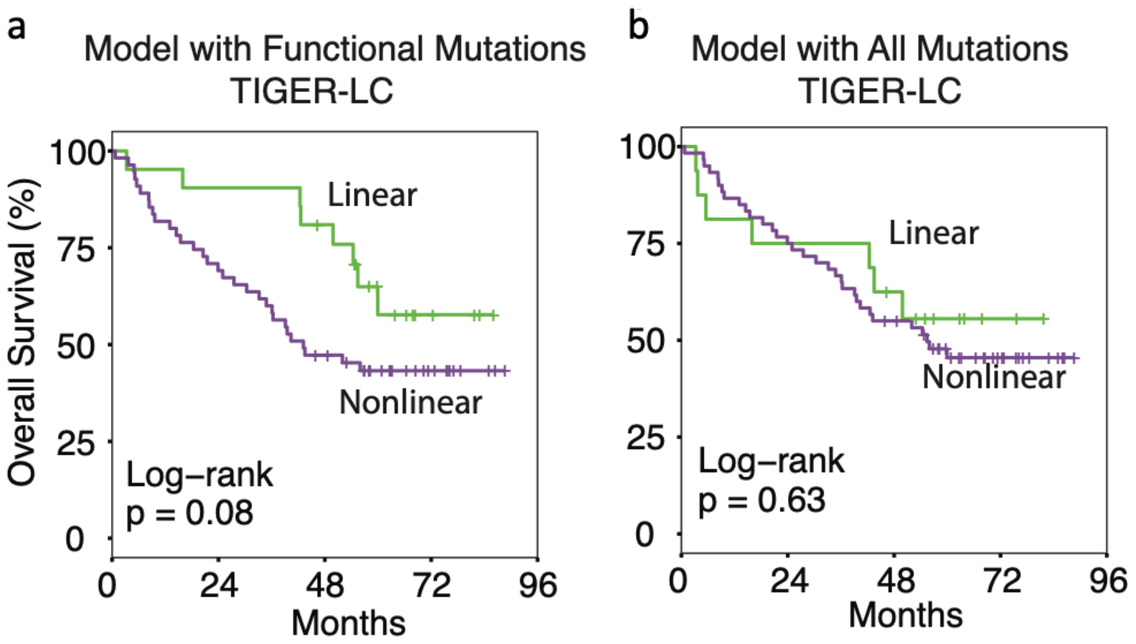

Supplementary Figure 1. Functional clonality reveals survival differences of linear versus nonlinear trees. (a-b) Kaplan Meier curves for the linear versus nonlinear trees for TIGER-LC cohort resulting from clonality analysis with only functional mutations and with all mutations respectively. We performed a stratified (by cohort) log-rank test to test if there is a difference in linear versus non-linear tree type. We found a significant p-value of 0.00001.

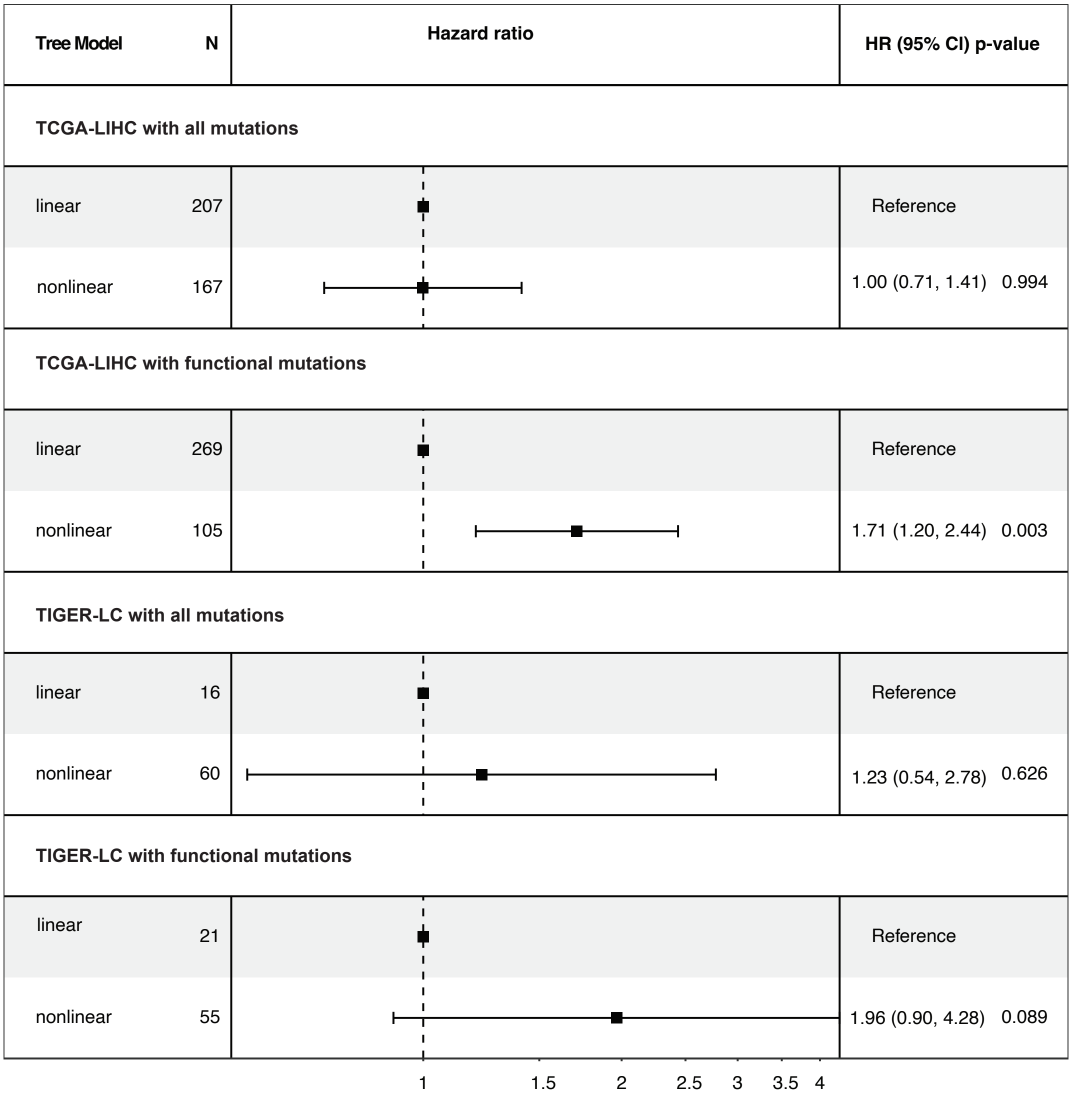

Supplementary Figure 2. Functional clonality reveals hazard ratio differences of linear versus nonlinear trees. Forest plots showing the hazard ratios with 95% confidence intervals for the hazard ratio of linear versus nonlinear trees for TCGA-LIHC and TIGER-LC cohorts resulting from clonality analysis with only functional mutations and with all mutations respectively.

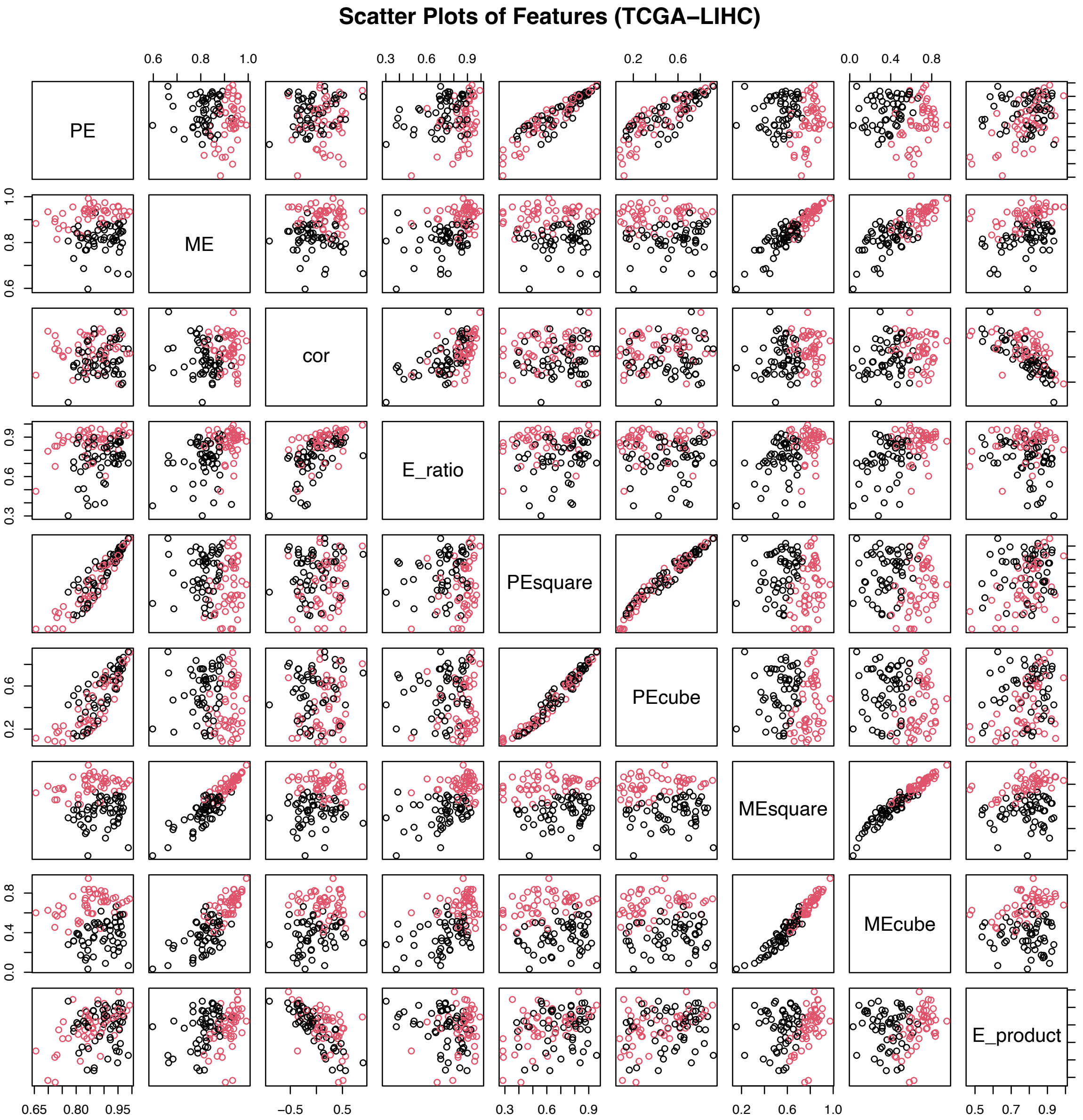

Supplementary Figure 3. Scatter plots of created features with the colors representing shallow branching (black) and deep branching (red) clusters for TCGA-LIHC cohort. These features are explained with details in methods section of the paper.

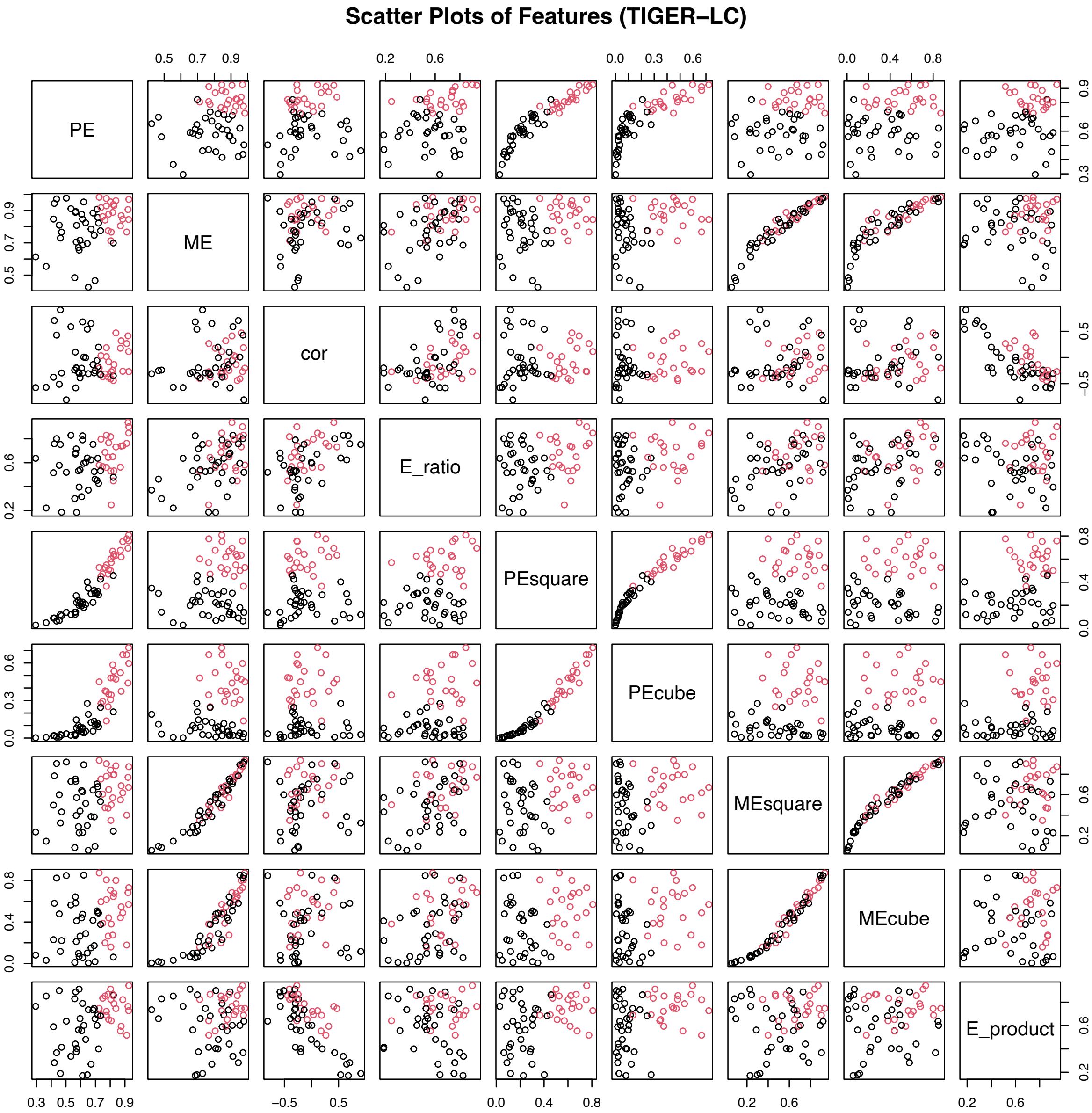

Supplementary Figure 4. Scatter plots of created features with the colors representing shallow branching (black) and deep branching (red) clusters for TIGER-LC cohort. These features are explained with details in methods section of the paper.

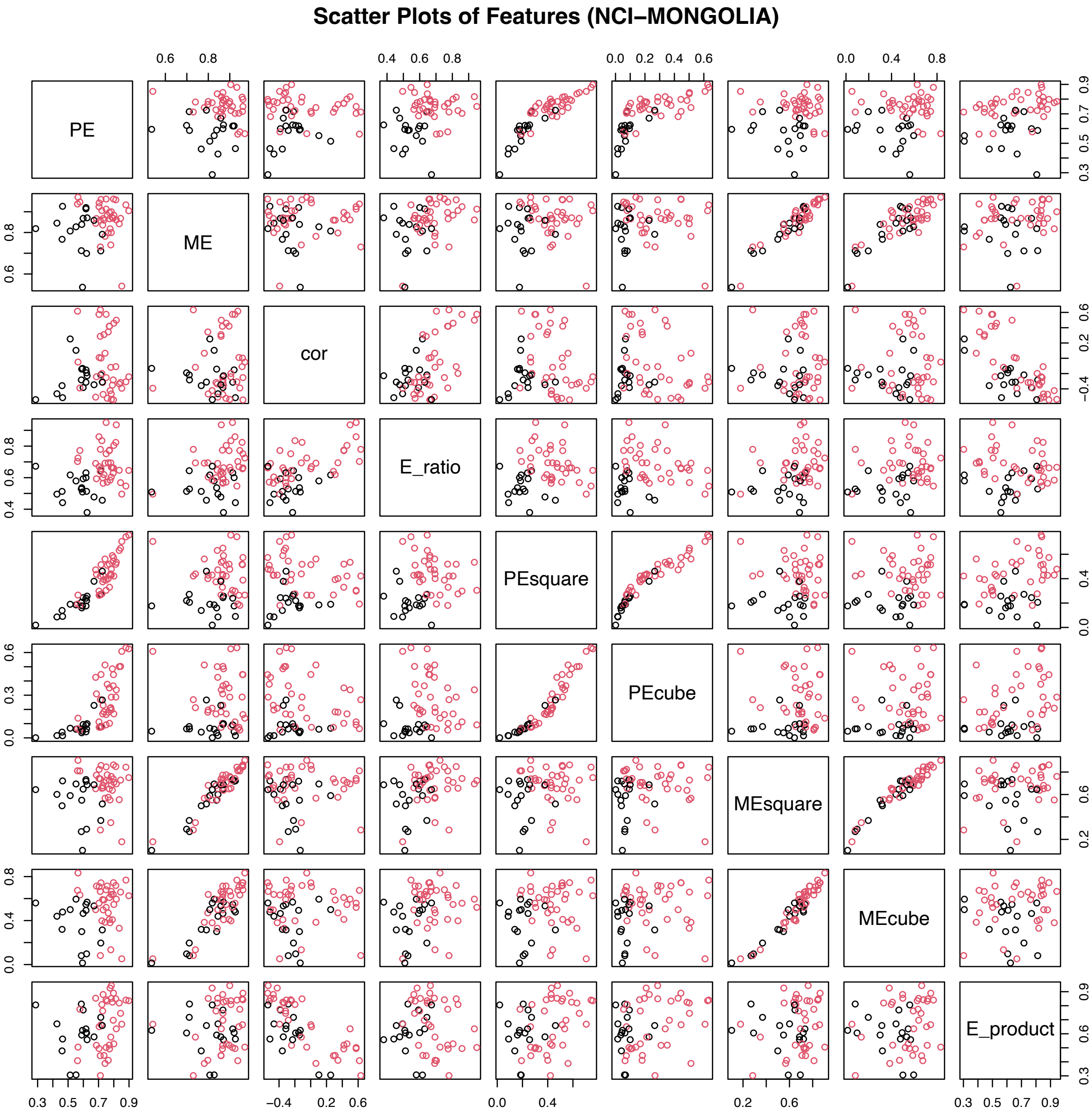

Supplementary Figure 5. Scatter plots of created features with the colors representing shallow branching (black) and deep branching (red) clusters for NCI-MONGOLIA cohort. These features are explained with details in methods section of the paper.

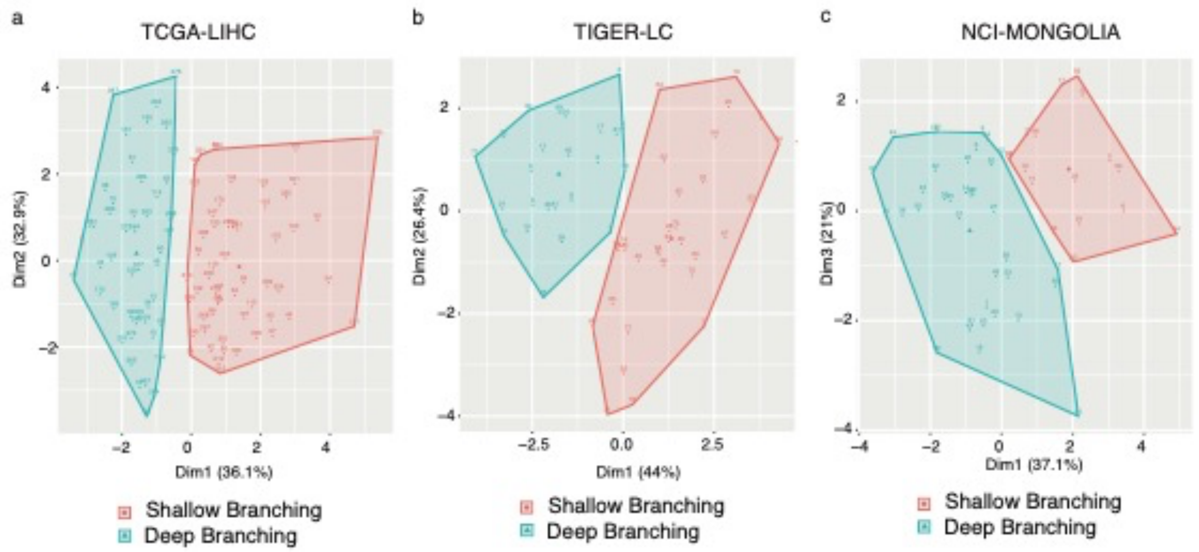

Supplementary Figure 6. (a) Plot of clustering results where x axis represents the first and y axis represents the second dimension of Principle Component Analysis (PCA) for TCGA-LIHC cohort. **(b)** Plot of clustering results where x axis represents the first and y axis represents the second dimension of Principle Component Analysis (PCA) for TIGER-LC cohort. **(c)** Plot of clustering results where x axis represents the first and y axis represents the third dimension of Principle Component Analysis (PCA) for NCI-MONGOLIA cohort.

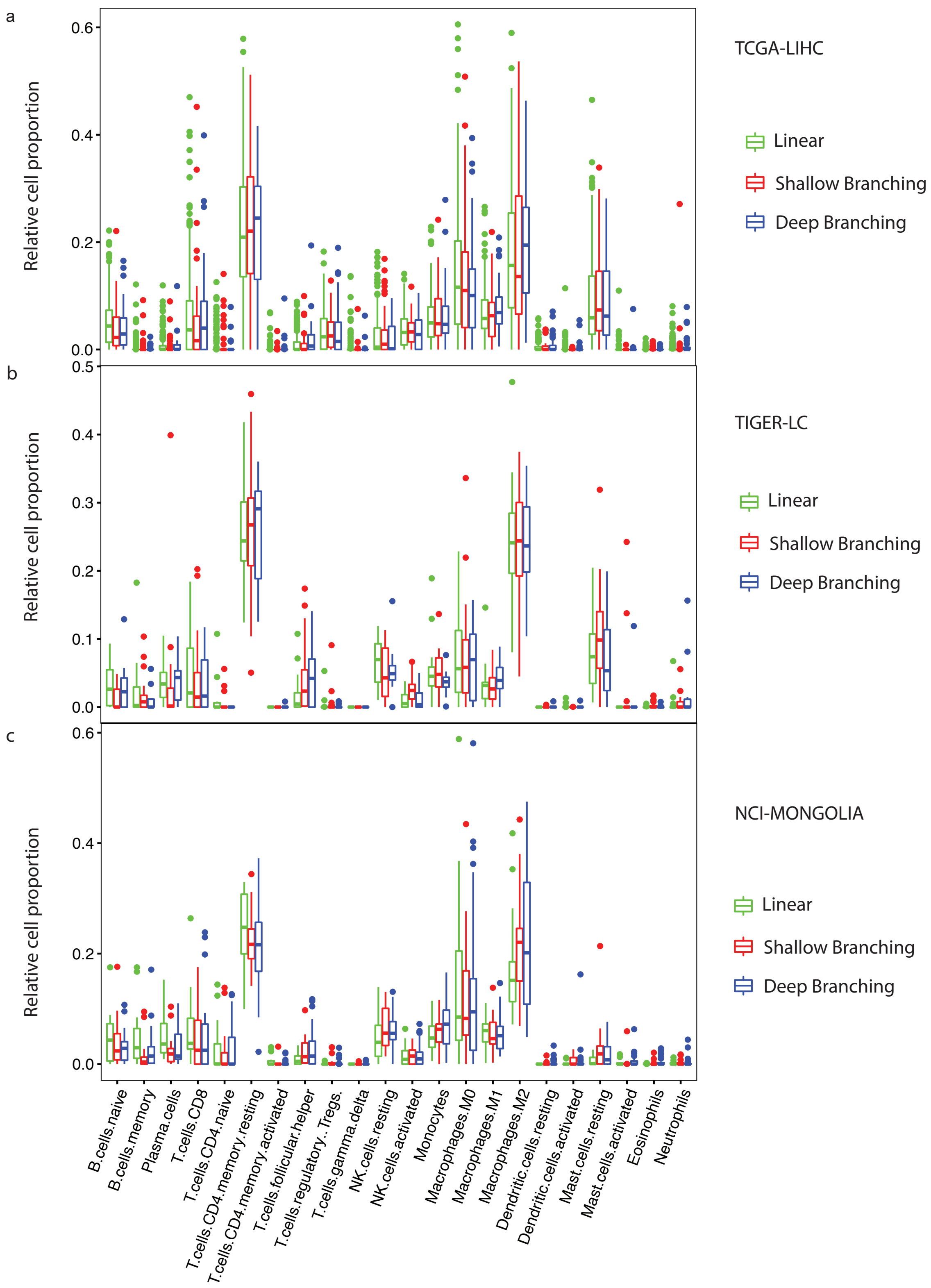

Supplementary Figure 7. Boxplots showing the relative cell proportions for each 22 immune cell type from CIBERSORTx with respect to tree phylogeny for **(a)** TCGA-LIHC, **(b)** TIGER-LC, and **(c)** NCI-MONGOLIA cohorts.

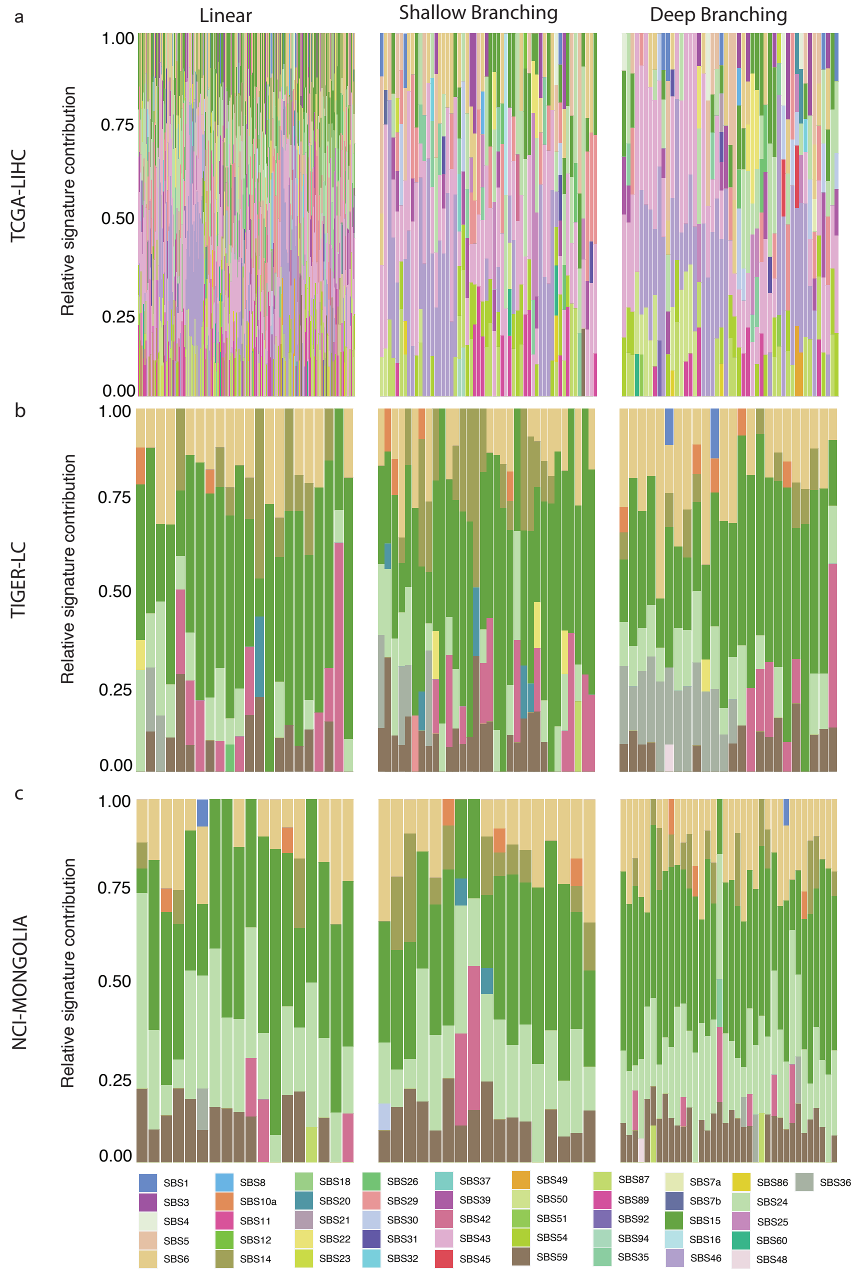

Supplementary Figure 8. (a) Stacked bar graphs showing the relative signature exposures for TCGA-LIHC cohort. Subject/signature weights were obtained from non-negative least squares mapping of individual samples (columns) vs reference signatures (rows) from the COSMIC catalogs24,25 and were also provided **(b)** Scatter plot of each pair of observations Euclidian distance versus proximity coming from a random forest model for TIGER-LC cohort. **(c)** Scatter plot of each pair of observations Euclidian distance versus proximity coming from a random forest model for NCI-MONGOLIA cohort.

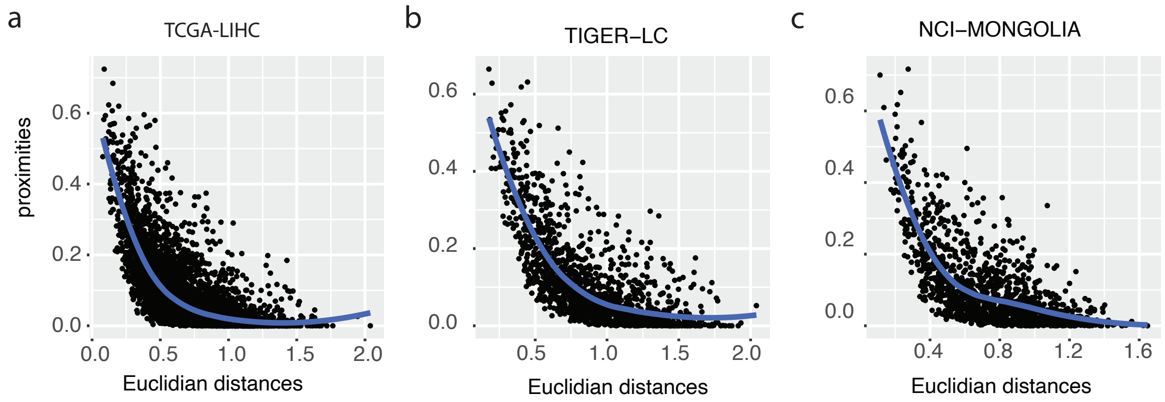

Supplementary Figure 9. (a) Scatter plot of each pair of observations Euclidian distance versus proximity coming from a random forest model for TCGGA-LHC cohort. **(b)** Scatter plot of each pair of observations Euclidian distance versus proximity coming from a random forest model for TIGER-LC cohort. **(c)** Scatter plot of each pair of observations Euclidian distance versus proximity coming from a random forest model for NCI-MONGOLIA cohort.

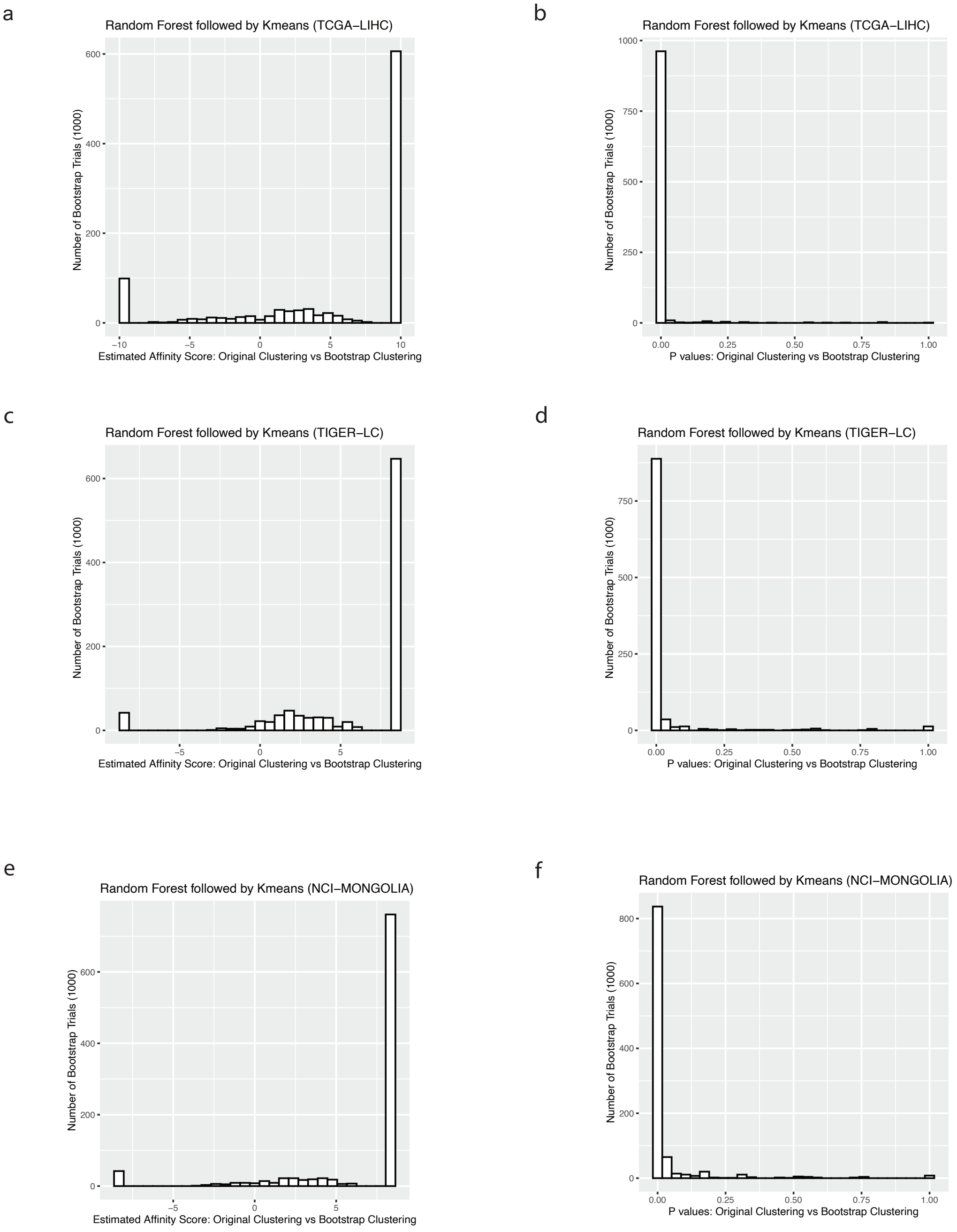

Supplementary Figure 10. (a,c,e) Histograms showing the estimated affinity score for 1000 bootstrap result for the cohorts TCGGA-LHC, TIGER-LC, and NCI-MONGOLIA respectively. **(b,d,f)** Histograms showing the two-sided p-values of the null hypothesis that affinity score is equal to zero for the cohorts TCGGA-LHC, TIGER-LC, and NCI-MONGOLIA respectively.

Supplementary Table 1. Clinical information of three HCC liver cancer cohorts involved in this study.

Variable	TCGA-LIHC (WEX:375, RNA:369)	TIGER-LC (WEX:78, RNA:51)	NCI-MONGOLIA (WEX:71, RNA:69)
**Age**			
$\leq 60\ y.o.$	194	20	33
$> 60\ y.o.$	180	56	37
NA	1	2	1
**Gender**			
$male$	254	59	36
$female$	121	17	34
NA	0	0	1
**Stage**			
$early$	261	23	19
$late$	90	10	35
NA	24	45	17
**Survival status**			
$alive$	244	37	49
$dead$	131	39	22

WEX: Whole-Exome sequencing. RNA: RNA sequencing.

Supplementary Table 2. Statistical comparisons of Age, Gender, and Stage across cohorts. Early stage (stage 1 and stage 2), and Late stage (stage 3 and stage 4). First column p-values are for the 2-sample t-Test for the mean age of individuals in given cohorts. Test statistics for TCGA-LIHC-TIGER-LC, TCGA-LIHC vs. NCI-MONGOLIA, and TIGER-LC vs. NCI-MONGOLIA comparisons are 2.36 (df=448), -0.25 (df=442), and -2.62 (df=144), respectively. Second column represent the 2-sample z-test for the proportion of female individuals in given cohorts. Third column represent the 2-sample z-test for the proportion of individuals whose tumor is in late stage (stage 3 or stage 4) in given cohorts.

Cohort Comparison	P-value for the mean comparison of AGE	P-value for the proportion comparison of female (GENDER)	P-value for the proportion comparison of late (STAGE)
TCGA-LIHC vs TIGER-LC	0.01	0.1	0.7
TCGA-LIHC vs NCI-MONGOLIA	0.8	0.01	<0.001
TIGER-LC vs NCI-MONGOLIA	0.02	0.001	0.004

Supplementary Table 3. Stratified Log Rank Test P-Values for Comparing Pairwise Phylogeny Clusters Stratified by Cohort. Each stratified log rank chi-square, assessing pairs of phylogeny groups treated as two-sample data stratified by cohort, is based on 1 degree of freedom.

	All cohorts stratified
Linear-Shallow	Chisq=1.3, p-value=0.3
Shallow-Deep	Chisq=10.4, p-value=0.001
Deep-Linear	Chisq=16.7, p-value<0.001
Linear-Nonlinear	Chisq=13.1, p-value<0.001

Supplementary Table 4. P-Values from Log Rank Tests Comparing Pairwise Phylogeny Clusters Across Cohorts. All p-values are calculated using 1-degree-of-freedom log rank chi-squares. These log rank tests are derived from standard two-group survival statistics, each based on pairs of phylogeny-group data treated as two-sample datasets.

Cohort	Linear vs Shallow Branching	Linear vs Deep Branching	Shallow Branching vs Deep Branching	Linear vs Nonlinear (Deep and Shallow Branching)
TCGA-LIHC	0.51	<0.001	0.007	0.03
TIGER-LC	0.57	0.009	0.04	0.08
NCI- MONGOLIA	0.3	0.4	0.9	0.3

Supplementary Table 5. 2-Sample Welch t-Test for Comparing Mean Mutations Between Linear and Shallow Branching Phylogeny Populations, Assuming Independence. Test statistic for the comparison of Linear vs Shallow Branching Tree Types for TCGA-LIHC, TIGER-LC, and NCI-MONGOLIA are -2.72, -2.02, and -1.74 respectively.

	TCGA-LIHC	TIGER-LC	NCI-MONGOLIA
Linear vs Shallow Branching	<0.001	0.04	0.09

Supplementary Table 6. Test for Independence of Phylogeny Type vs. Cancer Stage for TCGA-LIHC cohort: Pearson's Chi-squared Test (2 df) for 3 x 2 Contingency Table.

**TCGA-LIHC**	Early	Late
Linear	195	61
Shallow Branching	36	16
Deep Branching	30	13
	Pearson`s Chi-squared test p-value=0.44 for row-column independence	

Supplementary Table 7. Test for Independence of Phylogeny Type vs. Cancer Stage for TIGER-LC cohort: Fisher`s Exact test for 3x2 Contingency Table.

**TIGER-LC**	Early	Late
Linear	7	2
Shallow Branching	5	4
Deep Branching	11	4
	Fisher`s exact test p-value=0.63	

Supplementary Table 8. Test for Independence of Phylogeny Type vs. Cancer Stage for NCI-MONGOLIA cohort: Fisher`s Exact test for 3x2 Contingency Table.

**NCI-MONGOLIA**	Early	Late
Linear	4	9
Shallow Branching	5	7
Deep Branching	10	19
	Fisher`s exact test p-value=0.84	

Supplementary Table 9. Analysis of Deviance Table for Cox Multiple Regression Model Results for TCGA-LIHC cohort (Phylogeny type, Stage, Age and Gender). Here, the significant of each coefficient is tested in the present of all other variables.

TCGA-LIHC: Analysis of Deviance Table (Type II tests)		
N=350 (117 dead)	LR Chisq	P-value
Phylogeny Type	9.87	0.0077
Stage	17.84	<0.0001
Age	3.23	0.0722
Gender	0.05	0.8318

Supplementary Table 10. Analysis of Deviance Table for Cox Multiple Regression Model Results for TIGER-LC cohort (Phylogeny type, Stage, Age and Gender). Here, the significant of each coefficient is tested in the present of all other variables.

TIGER-LC: Analysis of Deviance Table (Type II tests)		
N=33 (19 dead)	LR Chisq	P-value
Phylogeny Type	3.86	0.14
Stage	7.85	0.005
Age	0.41	0.52
Gender	0.001	0.97

Supplementary Table 11. Analysis of Deviance Table for Cox Multiple Regression Model TIGER-LC cohort (Phylogeny type, Age and Gender). Here, the significant of each coefficient is tested in the present of all other variables.

TIGER-LC: Analysis of Deviance Table (Type II tests)		
N=76 (39 dead)	LR Chisq	P-value
Phylogeny type	7.50	0.02
Age	0.11	0.73
Gender	0.93	0.33

Supplementary Table 12. Analysis of Deviance Table for Cox Multiple Regression Model for NCI-MONGOLIA cohort (Phylogeny type, Stage, Age and Gender). Here, the significant of each coefficient is tested in the present of all other variables.

NCI-MONGOLIA: Analysis of Deviance Table (Type II tests)		
N=54 (17 dead)	LR Chisq	P-value
Phylogeny type	3.25	0.2
Stage	4.56	0.03
Age	0.02	0.89
Gender	2.26	0.13
