# Supplementary material for: Characterization of tumor evolution by functional clonality and phylogenetics in hepatocellular carcinoma
Source: Commun Biol. 2024 Mar 29;7:383. doi: 10.1038/s42003-024-06040-9 (PMC11245610; doi:10.1038/s42003-024-06040-9)
Supplement: Supplementary file 3 — Description of Additional Supplementary Files [file 42003_2024_6040_MOESM3_ESM.pdf]

Description of Additional Supplementary Files

File name: Supplementary Data 1

Description: Information about the functional genes used in this study.

File name: Supplementary Data 2

Description: Permutation test p-values for comparison of linear versus nonlinear tumor evolution trees
