# Supplementary material for: Characterization of tumor evolution by functional clonality and phylogenetics in hepatocellular carcinoma
Source: Commun Biol. 2024 Mar 29;7:383. doi: 10.1038/s42003-024-06040-9 (PMC11245610; doi:10.1038/s42003-024-06040-9)
Supplement: Supplementary file 6 — Reporting summary [file 42003_2024_6040_MOESM6_ESM.pdf]

nature portfolio

Corresponding author(s): COMMSBIO-23-1474-T

Last updated by author(s): Jan 18, 2024

Reporting Summary

Nature Portfolio wishes to improve the reproducibility of the work that we publish. This form provides structure for consistency and transparency in reporting. For further information on Nature Portfolio policies, see our Editorial Policies and the Editorial Policy Checklist.

Statistics

For all statistical analyses, confirm that the following items are present in the figure legend, table legend, main text, or Methods section.

n/a	Confirmed	
☐	☒	The exact sample size (*n*) for each experimental group/condition, given as a discrete number and unit of measurement
☐	☒	A statement on whether measurements were taken from distinct samples or whether the same sample was measured repeatedly
☐	☒	The statistical test(s) used AND whether they are one- or two-sided<br>*Only common tests should be described solely by name; describe more complex techniques in the Methods section.*
☐	☒	A description of all covariates tested
☐	☒	A description of any assumptions or corrections, such as tests of normality and adjustment for multiple comparisons
☐	☒	A full description of the statistical parameters including central tendency (e.g. means) or other basic estimates (e.g. regression coefficient) AND variation (e.g. standard deviation) or associated estimates of uncertainty (e.g. confidence intervals)
☐	☒	For null hypothesis testing, the test statistic (e.g. *F*, *t*, *r*) with confidence intervals, effect sizes, degrees of freedom and *P* value noted<br>*Give P values as exact values whenever suitable.*
☒	☐	For Bayesian analysis, information on the choice of priors and Markov chain Monte Carlo settings
☐	☒	For hierarchical and complex designs, identification of the appropriate level for tests and full reporting of outcomes
☐	☒	Estimates of effect sizes (e.g. Cohen's *d*, Pearson's *r*), indicating how they were calculated

Our web collection on statistics for biologists contains articles on many of the points above.

Software and code

Policy information about availability of computer code

Data collection	Public expression dataset were downloaded from the GEO data repository under the accession number GSE144269 and from TCGA portal (https://portal.gdc.cancer.gov).
Data analysis	We used R software to analyze data. *GraphPad Prism 7 and SPSS Statistics Subscription version 1.0.0.1089 (IBM) for statistic analysis, BRB-Array Tools version 4.6.0 for identifying differential expressed genes and feature selection, GENESIS V1.8.1 (Institute for Genomics and Bioinformatics, Graz University of Technology) for hierarchical clustering analysis and the generation of heatmap, Ingenuity Pathway Analysis (QIAGEN Bioinformatics) for pathway analysis, Nexus 9.0 (BioDiscovery) for copy number variation analysis. The results of flow cytometry was analyzed using FlowJo 10. ATAC-seq data process and peak calling were performed using Cutadapt v1.18, Bowtie2 V2.2.6, picard v2.18.26 and Generich (https://github.com/jsh58/Genrich). Subsequent downstream analysis after peak calling were carried out using DiffBind v3.9, ChIPseeker v3.9, i-cisTarget (https://gbiomed.kuleuven.be/apps/lcb/i-cisTarget/), IGV v2.7.x and deepTools2.*

For manuscripts utilizing custom algorithms or software that are central to the research but not yet described in published literature, software must be made available to editors and reviewers. We strongly encourage code deposition in a community repository (e.g. GitHub). See the Nature Portfolio guidelines for submitting code & software for further information.

Data

Policy information about availability of data

All manuscripts must include a data availability statement. This statement should provide the following information, where applicable:

- Accession codes, unique identifiers, or web links for publicly available datasets
- A description of any restrictions on data availability
- For clinical datasets or third party data, please ensure that the statement adheres to our policy

The processed whole exome sequencing data. The publicly available datasets used in this study include the TCGA database (TCGA-LIHC) [https://portal.gdc.cancer.gov/projects/TCGA-LIHC]. For NCI-MONGOLIA cohort, RNA Sequencing data are available at the Gene Expression Omnibus (GEO) repository under Study Accession GSE144269, and Whole-Exome Sequencing data are available at the dbGaP repository under Study Accession phs002000.v1.p1.
TIGER-LC data available at the dbGap repository under Study Accession phs001199.v2.p1.

Human research participants

Policy information about studies involving human research participants and Sex and Gender in Research.

Reporting on sex and gender	Not applicable
Population characteristics	This study does not introduce new HCC cohorts. For the published HCC cohorts, the age, gender, clinical stage, and other clinical data of each HCC cohorts were as previously reported in the references: Candia et al., Nat Commun 2020, 11(1), 4383, Chaisaingmongkol et al., Cancer Cell 2017, 32(1), 57-70 e3, and The Cancer Genome Atlas Research Network, Cell 2017, 169(7), 1327-1341 e23.
Recruitment	For HCC cohorts, the detail recruitment methods and potential selection bias were detailed as previously reported (Candia et al., Nat Commun 2020, 11(1), 4383, Chaisaingmongkol et al., Cancer Cell 2017, 32(1), 57-70 e3, The Cancer Genome Atlas Research Network, Cell 2017, 169(7), 1327-1341 e23).
Ethics oversight	For HCC cohorts, detail information regarding patient informed consent and ethical review were reported in Candia et al., Nat Commun 2020, 11(1), 4383, Chaisaingmongkol et al., Cancer Cell 2017, 32(1), 57-70 e3.

Note that full information on the approval of the study protocol must also be provided in the manuscript.

Field-specific reporting

Please select the one below that is the best fit for your research. If you are not sure, read the appropriate sections before making your selection.

☒ Life sciences ☐ Behavioural & social sciences ☐ Ecological, evolutionary & environmental sciences

For a reference copy of the document with all sections, see nature.com/documents/nr-reporting-summary-flat.pdf

Life sciences study design

All studies must disclose on these points even when the disclosure is negative.

Sample size	There was no sample size calculation. We used available samples in the studies. This is an exploratory study that integrated high throughput scRANseq and global tumor transcriptome in HCC, and no statistical methods were used to predetermine sample size. The total number of samples includes 675 HCC tumors, 464 non-tumor liver tissues and 10175 cells analyzed by scRANseq. We included all data from each cohort for corresponding analysis.
Data exclusions	No data was excluded from analysis.
Replication	All the results related to in vitro and in vivo experiments resulted from observations performed in three or more independent experiments. All attempts at replication of these experiments were successful.
Randomization	Method of randomization were not required in this study because samples and corresponding controls were processed at the same time and there were no significant differences between groups at the time of intervention.
Blinding	This is not a study involving clinical trial, blinding dose not apply to this study.

Reporting for specific materials, systems and methods

We require information from authors about some types of materials, experimental systems and methods used in many studies. Here, indicate whether each material, system or method listed is relevant to your study. If you are not sure if a list item applies to your research, read the appropriate section before selecting a response.

Materials & experimental systems

n/a	Involved in the study
☒	☐ Antibodies
☒	☐ Eukaryotic cell lines
☒	☐ Palaeontology and archaeology
☒	☐ Animals and other organisms
☒	☐ Clinical data
☒	☐ Dual use research of concern

Methods

n/a	Involved in the study
☒	☐ ChIP-seq
☒	☐ Flow cytometry
☒	☐ MRI-based neuroimaging
